# Metallicity, Atomic Disorder, and Li-Ion Storage in Fast-Charging Anodes

**DOI:** 10.1021/jacs.5c06578

**Published:** 2025-09-02

**Authors:** Kira E. Wyckoff, Arava Zohar, Tianyu Li, Yucheng Zhou, Linus Kautzsch, Welton Wang, Ananya Kepper, Ashlea R. Patterson, H. Cein Mandujano, Krishna Prasad Koirala, Anna Kallistova, Wenqian Xu, Jue Liu, Laurent Pilon, Anthony K. Cheetham, Ram Seshadri

**Affiliations:** † Materials Department and Materials Research Laboratory 8786 University of California, Santa Barbara, California 93106, United States; ‡ Mechanical and Aerospace Engineering Department Henry Samueli School of Engineering and Applied Science, 8786 University of California, Los Angeles, California 90095, United States; § Department of Chemistry and Biochemistry University of Maryland, College Park, Maryland 20742, United States; ∥ Advanced Photon Source, 1291 Argonne National Laboratory Lemont, Lemont, Illinois 60439, United States; ⊥ Neutron Scattering Division, 6146 Oak Ridge National Laboratory, Oak Ridge, Tennessee 37831, United States; # Department of Chemistry and Biochemistry, 8786 University of California, Santa Barbara, California 93106, United States

## Abstract

Oxides of Nb with Wadsley-Roth shear structures comprise a family of stable, high-rate anode materials for Li-ion batteries. A particular pair of them offers the unusual opportunity to test how important metallic conduction of the starting electrode is for electrode performance. The selected pair of compounds with similar 4 × 3 Wadsley-Roth block structures are insulating Ti_2_Nb_10_O_29_ and metallic Nb_12_O_29_. A combination of diffraction, electrochemistry, magnetic measurements, and entropic potential measurements is employed to establish key findings for these two anode materials. We find that starting with a metallic oxide is not especially advantageous over a comparable material that readily transitions into a metallic state upon lithiation. Second, the rate performance appears to be dictated by ion mobility, and atomic Ti/Nb disorder in Ti_2_Nb_10_O_29_ contributes to improved capacity retention at high rates by suppressing Li-ion ordering. However, subtle details in the nature of redox processes make Nb_12_O_29_ a slightly better electrode material for long-term cycling at slower rates.

## Graphical abstract

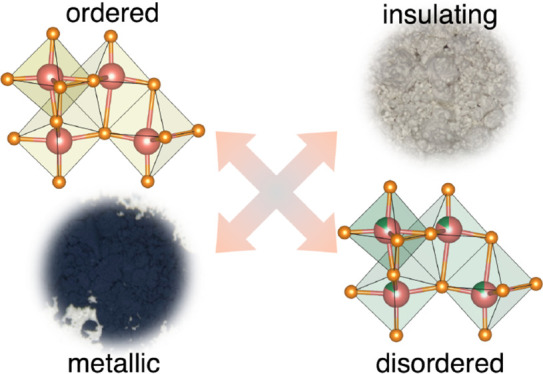

## Introduction

The worldwide transition to electric mobility has highlighted the potential of Li–ion batteries as an enabling and replacement technology for gasoline-powered internal combustion engines.^1–3^ As electric and hybrid vehicles become popular, the charging speed of Li–ion batteries has become an important consideration. The need to charge Li–ion batteries to high capacity in less than 15 min is a critical use case that must be solved. This threshold ultimately depends on many cell and pack-level details, but the electrode materials themselves dictate the limits of capacity storage and cycling rates.^4,5^ Ideally, Li metal would make the best anode in terms of energy density, but it comes with significant challenges.^6^ Graphite is the most commonly used anode material owing to its affordability and relatively stable performance.^2^ However, graphite has several drawbacks relating to slow Li insertion and safety concerns associated with the low redox voltage.^7^ These limitations have led to increased exploration and study of other anode material candidates with higher capacities, improved ion diffusion, and superior safety features.

Oxides of Nb with Wadlsey-Roth block structures represent a promising family of materials for Li–ion battery anodes.^8–23^ These materials consist of early transition metals that undergo multielectron redox, significantly increasing energy density.^24^ Their structures derive from ReO_3_-type corner-sharing blocks that are separated by crystallographic shear planes of edge-sharing octahedra. The compounds are primarily Nb or Nb–rich oxides. Edge-sharing octahedra along the shear planes enables good electron transport and adds structural rigidity.^25^ Corner-sharing octahedra separate these shear planes and form optimally sized channels for facile Li–ion transport.^26^ Electrochemical characterization of many Wadsley-Roth compounds have revealed high capacity retention at slow rates. At elevated rates, these materials cycle one electron *per* Nb or less, although, in theory, they can accommodate two electrons since both Nb^4+^ and Nb^3+^ are accessible.

Low electrode electronic conductivity is known to inhibit battery performance due to increased resistance during cycling.^27^ Many Wadsley-Roth compounds and other oxides that are used as electrodes are insulators, at least prior to lithiation. Several different approaches have been taken to enhance conductivity in insulating electrode materials, such as conductive additives^28^ or increasing particle surface area,^29^ including engineering nanofibers^30,31^ and nanospheres^32^ to reduce the path for electron transport. Here, we examine how insulator-to-metal transitions during Li insertion contribute to the conductivity. Griffith et al.^17^ and Preefer et al.^33^ showed that insulator-to-metal transitions are observed in some Wadsley-Roth electrode materials upon lithiation. To better understand the contribution of such a transition to electrical conductivity and electrode performance, we selected a model system of two compounds that share the same structure, where one is an insulator and the other is a metal.

Ti_2_Nb_10_O_29_ and Nb_12_O_29_ have 4 × 3 block structures, but the former has completely empty d levels and is insulating, while the latter has two d electrons distributed over the 12 Nb sites and is metallic with an antiferromagnetic ground state. The metallic (and low-temperature magnetic) nature of Nb_12_O_29_ has been established over several decades experimentally,^34–36^ and also from first-principles electronic structure calculations.^37^ Another difference is the degree of cation order and symmetry between the two systems as a result of the substitution of Nb with Ti in Ti_2_Nb_10_O_29_.^39^ Comparison of these materials should pinpoint the impact of initial metallicity and insulator-to-metal transition during lithium insertion,^33^ giving direct insight into the importance of dis/order on electrochemical processes. Other parameters can also affect cycling performance in these materials, such as the block size, Li–ion ordering at specific sites,^40^ and elemental composition. However, we intentionally selected this model system to reduce effects from those parameters.

Nb_12_O_29_ and Ti_2_Nb_10_O_29_ have been individually studied and show promising performance as anode materials,^10,15,41,42^ but have never been directly compared. Our results show that both materials display similar performance at slow rates. However, at faster cycling rates, Ti_2_Nb_10_O_29_ shows higher capacity retention compared to Nb_12_O_29_. The insertion kinetics, including ion diffusion, Li–ion ordering, and electrochemical redox were individually evaluated for their impact on rate performance. The results show the same order of magnitude for Li–ion transport in both materials. The measurements indicate increased Li–ion ordering in Nb_12_O_29_ over Ti_2_Nb_10_O_29_. Ti_2_Nb_10_O_29_, with a lower propensity for Li–ion ordering, showed improved Li insertion kinetics, demonstrating higher capacity at fast rates. This concept of Li-site preferences has been noted before, where high site preferences can add energetic barriers to Li insertion. By reducing energetic barriers related to Li–ion ordering, it is possible to improve capacity at faster cycling rates.^43^ Long-term cycling results show a greater capacity loss for Ti_2_Nb_10_O_29_ compared to Nb_12_O_29_. Ex situ XPS shows a large amount of Nb^3+^ in Ti_2_Nb_10_O_29_, suggesting a possible pathway to decomposition. In this study, we demonstrate transition metal disorder as a design strategy for fast-charging electrode materials and discuss the limits of Nb redox in shear structures.

## Methods

### Preparation of Nb_12_O_29_ and Ti_2_Nb_12_O_29_

To prepare Ti_2_Nb_10_O_29_ and Nb_12_O_29_, the appropriate amount of powders of TiO_2_ (Aldrich Chemical Co., 99%), Nb_2_O_5_ (Materion, 99.95%), and niobium metal were ground in an agate mortar for 20 min, pressed into 1 g pellets to 4.0 tons in a 13 mm die, and placed into a 10 mL alumina crucible.^44^ The 10 mL crucible was placed inside of a furnace at 1100 °C for 24 h. For Nb_12_O_29_ and all samples containing reduced Nb, pellets were placed in an vitreous silica tube, evacuated three times, and filled with 40 mmHg partial pressure of Ar.^24^ Following sealing, the tube was placed in a furnace and heated at 1100 °C for 24 h. Both materials were immediately air quenched after heating, allowed to cool, and reground to lose powder in an agate mortar for X-ray diffraction.

### X-ray and Neutron Diffraction

Powder X-ray diffraction (PXRD) measurements were performed using a Panalytical Empyrean powder diffractometer in reflection mode with a Cu *K*α radiation source. Rietveld analysis was performed to confirm the structure and phase purity using the TOPAS software package. Crystal structures were visualized using VESTA. High-resolution synchrotron X-ray powder diffraction (SXPD) data were collected at the 11-IDC beamline at the Advanced Photon Source (APS) at Argonne National Laboratory. A 2D PerkinElmer a-Si flat panel detector was used with an average wavelength of λ = 0.1173 Å. Samples were also examined using time-of-flight (TOF) neutron diffraction. The neutron diffraction patterns were collected at 300 K on the Nanoscale-Ordered Materials Diffractometer (NOMAD) at the Spallation Neutron Source (SNS) at Oak Ridge National Laboratory (ORNL). *Operando* synchrotron X-ray powder diffraction measurements were carried out at beamline 17-BM at the Advanced Photon Source at Argonne National Laboratory. All measurements were performed in AMPIX cells at room temperature in transmission geometry at a wavelength (λ = 0.458924 Å). The same Li foil counter electrode, and slurry cast composite ratio [80:10:10 (wt %) active:SuperP:PVDF] were used as previously described, with the slurry instead cast onto a Celgard separator. Cast electrodes weighed approximately 4 mg and were 16 mm in diameter. The AMPIX cell was assembled in an argon glovebox using lithium metal as the counter electrode, a glass fiber separator (Whatman), and 1.0 M LiPF_6_ dissolved in 1:1 v/v ethylene carbonate/diethyl carbonate (EC/DEC). The cell was cycled at a 1C rate for two complete charge/discharge cycles.

### Scanning Transmission Electron Microscopy

Samples for scanning transmission electron microscopy (STEM) were prepared by dispersing the powders onto lacey carbon-supported copper grids. STEM imaging was conducted using an aberration-corrected Thermo Fisher Scientific Spectra microscope operated at an accelerating voltage of 200 kV. High-angle annular dark-field (HAADF) imaging was performed with a convergence semiangle of 15 mrad, a collection angle range of 60–200 mrad, and a probe current of 41 pA to mitigate any beam-induced damage. Each HAADF-STEM image was generated by integrating 20 drift-corrected image frames to enhance signal-to-noise ratio and image stability using the Velox software from Thermo Fisher Scientific.

### Raman Spectroscopy

Raman spectroscopy was performed at room temperature using a Horiba Jobin Yvon T64000 open-frame confocal microscope operating at a wavelength of 488 nm with a monochromator and an LN_2_-cooled CCD array detector. Filters were used to reduce the laser to 50% of its original intensity to prevent beam damage to the samples. Spectra were calibrated by referencing the monocrystalline silicon spectrum, which peaks at 521 cm^–1^.

### Scanning Electron Microscopy

Powders were placed on double-sided carbon tape and inserted into an Apreo C FEG (ThermoFisher) microscope chamber. SEM images were collected using secondary electron (SE) and InLens detectors with a 10 keV accelerating voltage and a 0.8 nA current.

### Electrochemistry

Materials were cast onto a copper foil in a ratio of 8:1:1 of active material: conductive carbon (TIMCAL SuperP): polyvinylidene fluoride (Kynar 2800). The composite film was punched into 10 mm disk electrodes, and half cells were assembled in an argon-filled glovebox (MTI parts, 2032 SS casings) using polished lithium foil (Sigma-Aldrich) and 16 mm glass fiber separators (Whatman GF/D) and 1 M LiPF EC: DMC electrolyte. Cells were crimped at 0.8 tons of pressure. Electrochemistry experiments were performed on assembled coin cells using a BioLogic potentiostat VMP 3 (EC-Lab v11.43) at 25 °C in a controlled environment. To test extended cycling performance, galvanostatic cycling was performed at a cycling rate of 2C between 1 and 3 V for 350 cycles with a 5 min rest interval between cycles. For variable rate cycling, cycling rates of C/20, C/2, 1C, 2C, 5C, and 6C were used in that order. C rates were calculated based on one Li per transition metal. Data analysis was performed using Pandas in Python v3.9.12,^45^ and data plots were generated using Matplotlib.^46^

### Magnetic Measurements

Magnetic measurements were carried out using a Quantum Design MPMS3 SQUID magnetometer to measure magnetic susceptibility as a function of temperature. The samples were packed in an Ar-filled glovebox, approximately 5 mg each, and were measured in polypropylene capsules with air-free transfers of samples into the inert measurement chamber of the MPMS. DC susceptibility measurements were collected in both field-cooled warming (FCW) and zero-field-cooled warming (ZFCW) conditions with applied magnetic fields *H* ranging from *H* = 0.01 T to *H* = 5 T in the temperature range *T* = 2 K to *T* = 400 K.

### Potentiometric Entropy Measurements

The potentiometric entropy measurement technique was performed on coin cells with Nb_12_O_29_ and Ti_2_Nb_10_O_29_ working electrodes and lithium metal counter electrodes, using the setup described previously.^44,47,48^ Specifically, the open-circuit voltage *U*_*OCV*_(*x*, *T*) and entropic potential ∂*U*_*OCV*_(*x*, *T*)/∂*T* of coin cells were measured as functions of lithium composition *x*. Over the measurements at 20 °C, a series of 30 min constant current pulses at C/10 were imposed, each followed by a 270 min relaxation period. During the relaxation periods, a step-like temperature profile was applied to the coin cell from 15 to 25 °C in 5 °C increments with a thermoelectric cold plate (TE technology, CP-121). Simultaneously, the corresponding coin cell potential evolution was recorded with a potentiostat (BioLogic, VSP-300). Near the end of every temperature step, it was verified that the coin cell had reached thermodynamic equilibrium if (i) the temperature difference between the cold plate and the top of the coin cell was less than 0.1 °C and (ii) the time rate of change of the open-circuit voltage ∂*U*_*OCV*_/∂*t* was less than 1 mV h^–1^.

### X-ray Photoelectron Spectroscopy

The pristine Nb_12_O_29_ and lithiated samples extracted from coin cells for ex situ measurements were loaded onto an air-free sample holder in an Ar-filled glovebox, and then opened inside the XPS chamber under vacuum to minimize air exposure. Ti_2_Nb_10_O_29_ is completely oxidized and was loaded without the air-free holder. All powders were spread onto double-sided Scotch tape attached to a stainless steel sample holder. The samples were analyzed using a Thermo Fisher Escalab Xi+ XPS equipped with a monochromatic Al anode (*E* = 1486.7 eV). Survey scans were collected at a pass energy of 100 eV for 20 ms, averaged twice. High-resolution scans were collected in the Nb 3d region at a pass energy of 20 eV for 100 ms, averaged 5 times. All spectra were referenced to adventitious carbon at 284.8 eV. Fits were performed using CasaXPS, incorporating Shirley backgrounds and GL(50) peak shapes. The energy splitting associated with the d-orbital peaks resulted in two peaks, Nb 3d_5/2_ and Nb 3d_3/2_. The fit of the peak regions was locked to their corresponding 3:2 area ratios and constrained to have the same full width at half-maximum, with an energy gap determined as 2.78 eV.

## Results and Discussion

Nb_12_O_29_ and Ti_2_Nb_10_O_29_ materials are members of the Wadsley-Roth crystallographic family. The structures of both materials were characterized using a combination of synchrotron X-ray and neutron diffraction. Figure 1 displays the data and the results of a joint Rietveld refinent. The fit in Figure 1a for Nb_12_O_29_ shows good agreement with a crystal structure in the monoclinic *A*2/*m* space group. Figure 1c shows data for Ti_2_Nb_10_O_29_. In this case, the Rietveld refinement indicates a similar crystal structure with a different symmetry, the orthorhombic *Amma* space group. Most studies for these materials report monoclinic symmetry, which is impacted by synthesis conditions.^30–32,34,36,49,50^ Orthorhombic Ti_2_Nb_10_O_29_, with a mixture of two cations (Ti and Nb), increases configurational entropy by introducing disorder which enables higher symmetry, as reported for example, in halide perovskites.^51^ Although the two compounds have different symmetries, the structures are really quite similar. Based on X-ray diffraction of materials with varying amounts of Ti in Ti_*x*_Nb_12–*x*_O_29_ (*x* = 0.0, 0.5, 1.0, 1.5, and 2.0), we find that the unit cell volume decreases linearly as a function of the Ti amount *x* in the material, indicating a solid solution. The refinement of Ti_2_Nb_10_O_29_ was with Ti and Nb randomly distributed across the different sites. We note that Cheetham and Von Dreele^38^ have shown that Ti atoms favor replacing Nb in the periphery, along the shear planes rather than the center due to electrostatic effects.

**Figure 1:**
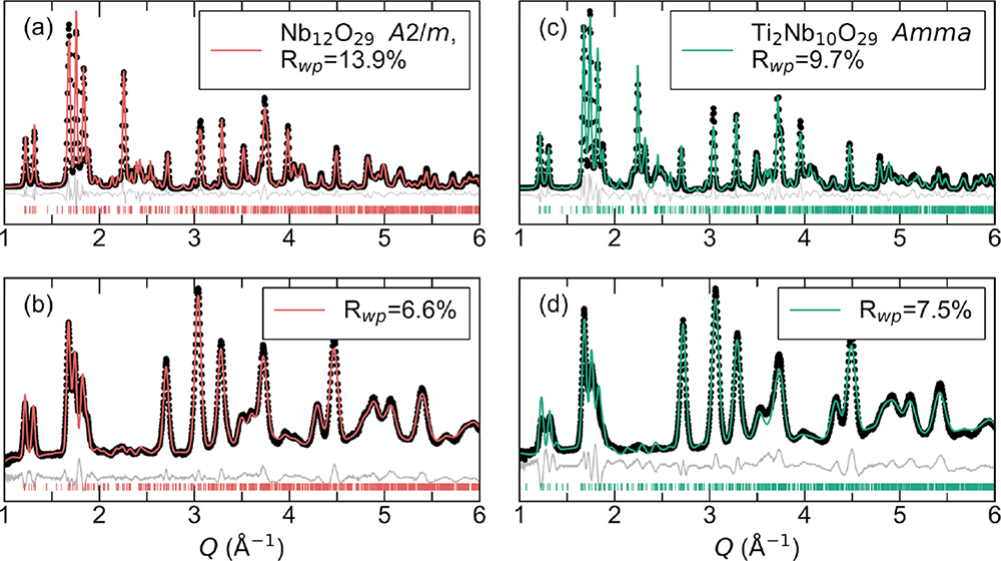
(a) Synchrotron powder X-ray diffraction and (b) neutron diffraction Rietveld refinement for Nb_12_O_29_. (c) Synchrotron powder X-ray diffraction and (d) neutron diffraction Rietveld refinement for Ti_2_Nb_10_O_29_. Diffraction data (circles) fit to data, and difference profiles are displayed. Synchrotron X-ray powder diffraction data were acquired at 11-IDC at the Advanced Photon Source, and neutron diffraction data were acquired at NOMAD at the Spallation Neutron Source.

The crystal structure of Nb_12_O_29_ is shown in Figure 2a. The structure comprises 3 × 4 ReO_3_-type blocks that tile in ribbons formed by overlapping edge sharing between the blocks at the same level (*a*–*bc* plane). One of these blocks is indicated with shading and an outline in Figure 2a. Figure 2b,c display the Li bond valence difference isosurface (Δν = 0.1 valence units) within two different projections of the crystal structure structure with bond valence maps displaying low-energy Li–ion diffusion paths in all directions. The orthorhombic crystal structure of Ti_2_Nb_10_O_29_ has the same block size and also tiles as ribbons along the *a*–c*b* plane, with effectively the same crystal structure.

**Figure 2:**
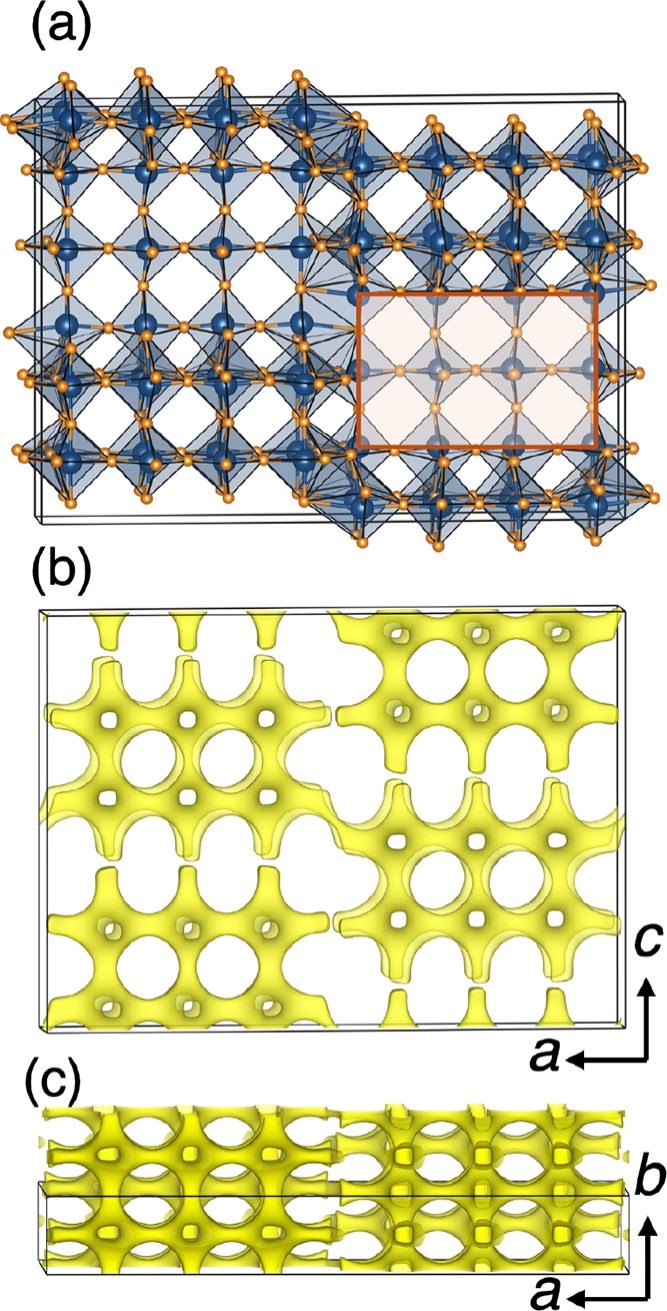
(a) Monoclinic crystal structure of Nb_12_O_29_ projected down the short *b* axis showing the 4 × 3 ReO_3_ blocks (one of the blocks highlighted and outlined) that are edge-connected with a shift along *b* and *c*. (b, c) Li bond valence difference isosurface (Δν = 0.1 valence units) created using softBV and projected down different directions of the crystal structure. Connected, low-energy pathways for Li^+^ diffusion are evident. The orthorhombic crystal structure of Ti_2_Nb_10_O_29_ is very similar to this depicted structure of Nb_12_O_29_.

The close similarity between the two crystal structures is further made clear from the HAADF-STEM imaging presented in Figure 3a where images of the different as-prepared compounds are displayed side-by-side. In addition to supporting the presence of very similar channels for Li–ion transport, the images also point to the absence of stacking faults and the relatively high degree of crystalline perfection, at least within these sections. A higher magnification image of Nb_12_O_29_ is displayed in Figure 3b and is compared with the projected position of Nb atoms in the monoclinic crystal structure.

**Figure 3:**
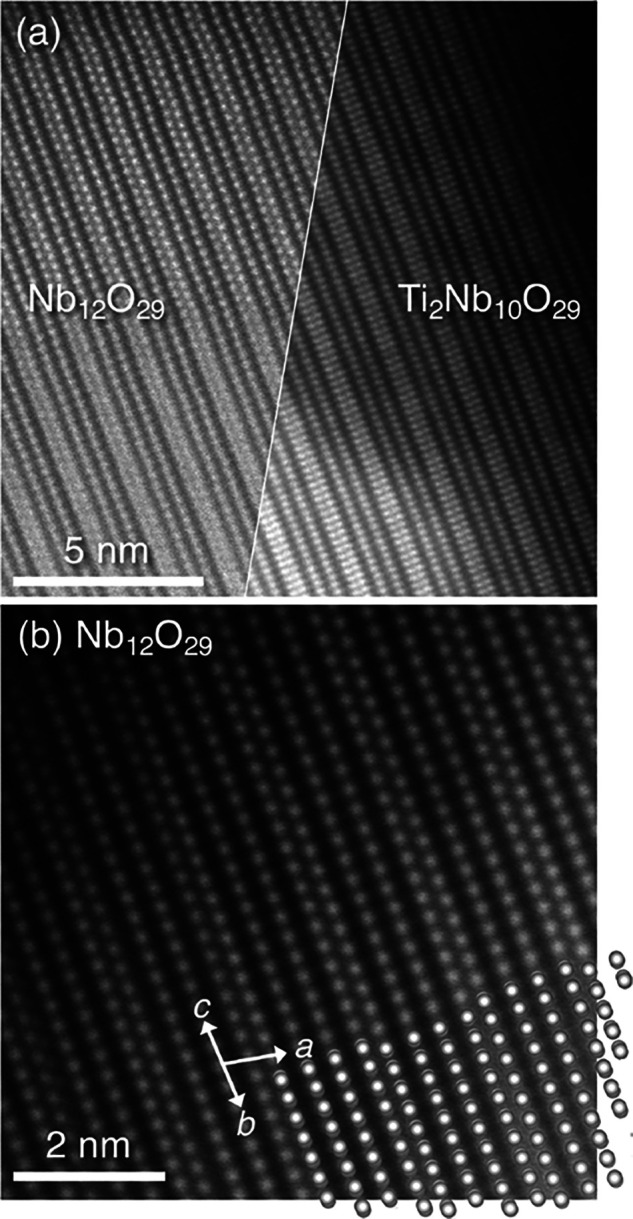
(a) STEM imaging of the crystal structures of monoclinic Nb_12_O_29_ and orthorhombic Ti_2_Nb_10_O_29_, emphasizing the close structural similarity. (b) Higher-resolution image of Nb_12_O_29_, shown with an overlay of an appropriate projection (axes indicated) of the Nb atom positions in the crystal structure.

Raman spectroscopy is an additional method to probe the crystallographic structure and bonding, as represented in the vibrational modes. Figure 4a shows that the high-frequency regime peaks for Nb_12_O_29_ are at 985, 633, and 460 cm^–1^, corresponding to Nb–O bond stretching modes of edge- and corner-sharing NbO_6_ octahedra. Low-frequency peaks, between 150 and 350 cm^–1^, suggest angle deformation bonds in descending bond order, such as metal–metal vibrations. All these peaks resemble stretching modes observed in the monoclinic Nb_2_O_5_ structure^52–55^ and are known to yield fewer peaks compared to Ti_2_Nb_10_O_29_. The Raman spectrum in Figure 4c shows the vibration modes of Ti_2_Nb_10_O_29_, with peaks located at 1001 and 894 cm^–1^, corresponding to corner- sharing octahedra and at 645 and 542 cm^–1^, corresponding to edge sharing *M*O_6_ (M = Ti or Nb) octahedra. Comparison of the Raman spectra shows that the vibration modes associated with the edge-sharing octahedra are more intense due to the contribution of Ti occupation in up to 40% of the octahedra.^52^ The peaks at 266 (262) and 222 (225) cm^–1^ represent distorted octahedra in both compounds. Generally, Raman spectra show good agreement with monoclinic and orthorhombic symmetries and support structural assignments from the X-ray and neutron diffraction data. Particle sizes of both materials were examined by scanning electron microscopy (SEM). The particle sizes of both materials are comparable, and are on the order of a few micrometers, as seen in Figure 4b,d. The characterization techniques discussed previously show subtle differences in symmetry between the two compounds with no structural connectivity or morphological differences that would significantly impact electrochemical performance.

**Figure 4:**
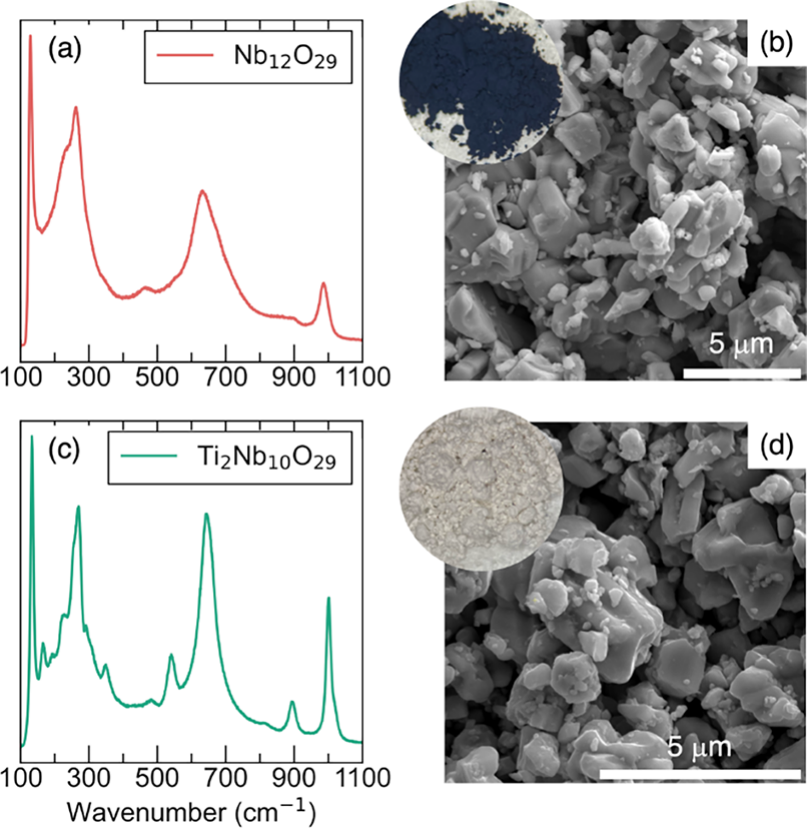
(a) Raman spectrum, (b) SEM image of Nb_12_O_29_ powders, (c) Raman spectra, and (d) SEM image of Ti_2_Nb_10_O_29_ powders. Raman data were collected with 488 nm laser excitation. The insets display the colors of the powders, which are bluish-black for metallic Nb_12_O_29_ and white for insulating Ti_2_Nb_10_O_29_.

The electrochemical performance associated with Li insertion for both materials were measured in a coin half-cell configuration vs Li metal with identical preparation protocols, as described in the methods section. Figure 5a shows the discharge and charge for the first cycle profile at the slow rate of C/10. The profile overlay of both materials shows significant capacity, approximately 260 mAh g^–1^ between 1 and 3 V. These materials display similar maximum capacity despite not containing the same amount of Nb. The theoretical capacity for Nb_12_O_29_ is 203.6 and 180 mAh g^–1^ for Ti_2_Nb_10_O_29_, assuming one electron per Nb atom for storage and no Ti redox. The two materials exhibit noticeable differences in the profiles of their discharge curves. Ti_2_Nb_10_O_29_ displays the characteristic shoulder plateau of Wadsley-Roth materials around 1.6 V, followed by a smooth and sloping curve. Nb_12_O_29_, on the other hand, has a shoulder plateau slightly above Ti_2_Nb_10_O_29_ and instead of a smooth discharge profile, the profile of Nb_12_O_29_ displays several clear kinks and plateaus.

**Figure 5:**
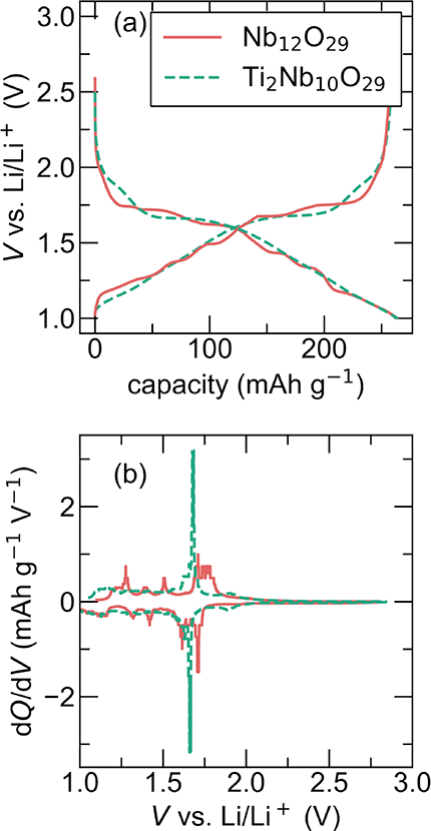
Electrochemistry of Nb_12_O_29_ and Ti_2_Nb_10_O_29_. (a) Galvanostatic cycling at a C/10 rate and (b) corresponding *dQ*/*dV vs V* plot to highlight the redox peaks. Note the smoother traces due to atomic disorder in Ti_2_Nb_10_O_29_, resulting in a single redox peak, in contrast to Nb_12_O_29_ which displays multiple peaks.

These peaks and differences are more prominent when evaluating *dQ*/*dV* traces in Figure 5b, where there is a clear single peak for Ti_2_Nb_10_O_29_ and many smaller peaks for Nb_12_O_29_. Based on the slow electrochemistry data, we can infer that the smooth profile of Ti_2_Nb_10_O_29_ is indicative of solid solution (except for the single flat region) during the insertion process, while the kinked profile of Nb_12_O_29_ indicates Li–ion ordering which is associated with two phase-coexistence regions.

Li insertion kinetics were examined using a variable rate cycling test and long-term cycling at a fast rate (Figure 6). The discharge profiles at different C rates are shown in Figure 6a,b for Nb_12_O_29_ and Ti_2_Nb_10_O_29_, respectively. The fast cycling curves are reminiscent of the slow cycling profiles, with a smooth discharge curve for Ti_2_Nb_10_O_29_ throughout the various rates, while Nb_12_O_29_ displays kinks in the discharge curve. As the applied current becomes larger and the cycling rate increases, the kinks become less prominent. Figure 6c summarizes the variable rate capacity retention. Increasing the cycling rate from C/20 to C/2 shows a clear bifurcation of capacities between the materials. Ti_2_Nb_10_O_29_ shows that it is capable of storing more charge than Nb_12_O_29_ at C rates of C/2, 1C, 2C, and 6C. Figure 6d demonstrates the long-term cycling of the materials at a 2C rate. These results show high capacity retention for Nb_12_O_29_, and capacity fade for Ti_2_Nb_10_O_29_, indicating a degradation process for the latter composition. This measurable difference in capacity retention at faster cycling rates was unexpected as both macroscopic particle size and structure are similar for both compounds. This begs the question, what is fundamentally driving improved rate performance in Ti_2_Nb_10_O_29_? To understand these results, it will be critical to understand any possible structural evolution, Li–ion diffusion, electronic conductivity, transition metal oxidation state, and Li–ion ordering differences between these materials.

**Figure 6:**
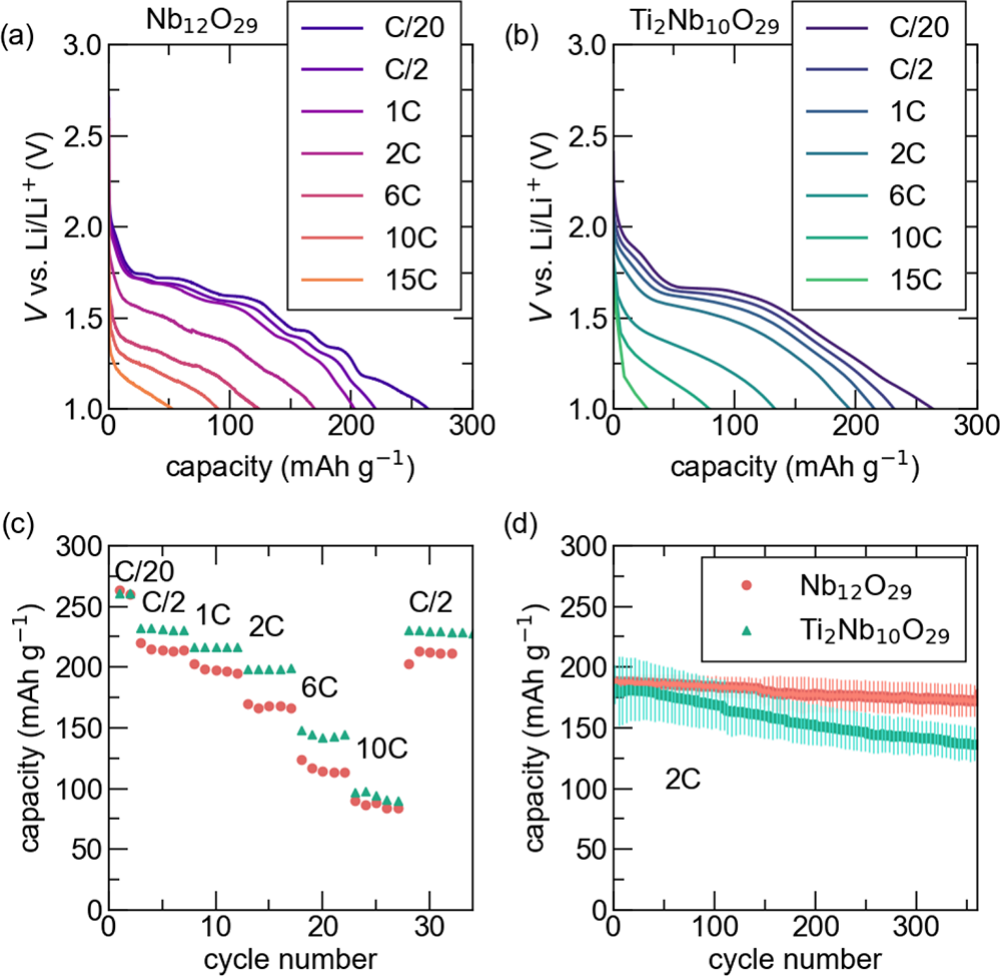
Galvanostatic charging at rates from C/10 to 10C for (a) Nb_12_O_29_ and (b) Ti_2_Nb_10_O_29_. (c) Summary of the capacity as a function of cycling rate for the two compounds. (d) Long-term cycling capacity between 1 and 3 V was performed at a rate of 2C following two formation cycles at a rate of C/10. Between 8 and 10 cells have been averaged.

*Operando* synchrotron diffraction data were collected to observe the structural evolution of Nb_12_O_29_ and Ti_2_Nb_10_O_29_ during lithium insertion and deinsertion. These data can provide insight into the nature of the structural transformations of these materials, including the reversibility of the transformation. Figure 7a,b show the corresponding electrochemistry, unit cell volume change, and diffraction heat map for Nb_12_O_29_ and Ti_2_Nb_10_O_29_, respectively. The data were collected for two complete discharge/charge cycles at a 1C cycling rate. X-ray diffraction data cannot probe Li atoms, and therefore, the data shows the bulk structural changes associated with transition metal octahedra. Both materials undergo reversible structural transformations based on the diffraction heat maps after each full cycle. Additionally, similar expansion/contraction crystallographic features are observed for both Nb_12_O_29_ and Ti_2_Nb_10_O_29_. Notably, we see in Figure 7a that Nb_12_O_29_ has a unit cell volume expansion of 6% whereas Figure 7b shows that Ti_2_Nb_10_O_29_ undergoes a volume change of approximately 4% but on the second cycle, reaches the same 6% change. The volume expansion in these structures occurs mainly along the [011] plane. These small volume changes are known for the Wadsley–Roth family, thanks to open channels and the ability to accommodate large amounts of Li–ions without changing the symmetry of the phase. The new phase that forms due to Li–ordering is clearly associated with low formation energy, enabling fast cycling. This has been previously seen, for example, in the highly rigid anode material LiScMo_3_O_8_.^56^

**Figure 7:**
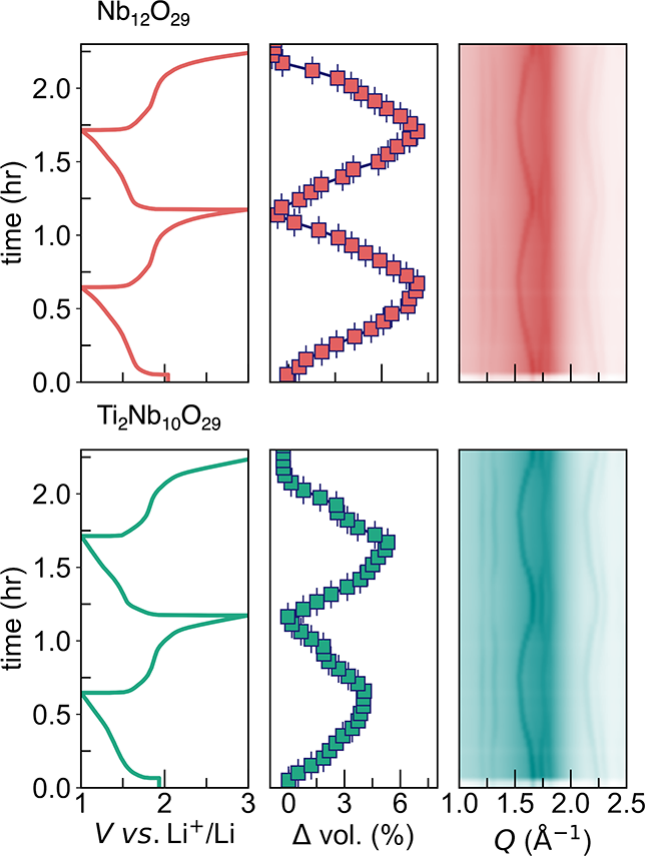
(from left to right) *Operando* electrochemistry data of (a) Nb_12_O_29_ and (b) Ti_2_Nb_10_O_29_ at a C/2 cycling rate, the volume change at each lithiation state with respect to the pristine electrode and the raw 17BM synchrotron diffraction for *Q* values between 1.0 and 2.5 A^–1^.

Magnetic susceptibility measurements were carried out on the pristine unlithiated parent materials Nb_12_O_29_ and Ti_2_Nb_10_O_29_, as well as three lithiated materials in the Li_*x*_Ti_2_Nb_10_O_29_ system. Figure 8a highlights temperature-dependent susceptibility data for all samples. As expected for a d^0^ transition metal oxide, Ti_2_Nb_10_O_29_ is an insulating diamagnet showing largely temperature-independent negative susceptibility. Susceptibility data displayed in Figure 8b for Nb_12_O_29_ shows local moment, paramagnetic behavior resulting from the two present *d* electrons for every formula unit. At lower temperatures, a transition to an antiferromagnetic state is observed as a change in slope, as reported previously.^34,36^ Ti_2_Nb_10_O_29_ samples show an interesting progression with increased lithiation. After inserting a small amount of Li in Li_4_Ti_2_Nb_10_O_29_, we see the onset of local-moment paramagnetic behavior. After inserting more Li at Li_7_Ti_2_Nb_10_O_29_, there is an increasing deviation from Curie–Weiss type paramagnetic behavior as a result of temperature-independent Pauli paramagnetism from delocalization of the introduced electrons. At full lithiation, for Li_15_Ti_2_Nb_10_O_29_, the susceptibility is dramatically different, showing largely temperature-independent Pauli paramagnetism, indicating good metallicity and very little local moment behavior.

**Figure 8:**
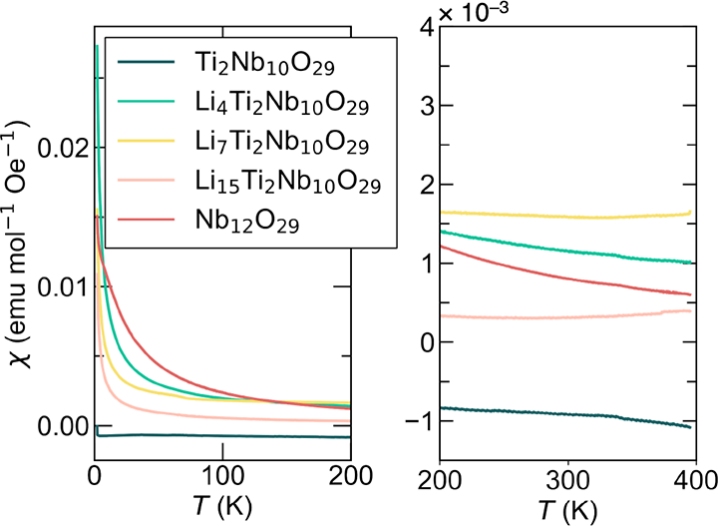
Magnetic susceptibility measurements of Nb_12_O_29_ and Li_*x*_Ti_2_Nb_10_O_29_ (*x* varying from 0.0 to 15) showing a combination of local moment magnetism and Pauli paramagnetism; the latter increasing with increasing *x* reflecting increasing metallic behavior.

There is no clear difference in volume expansion between Nb_12_O_29_ and Ti_2_Nb_10_O_29_, and to build a more robust picture, it is necessary to probe Li transport mechanisms and kinetics. Apparent Li–ion diffusion coefficients were measured using the galvanostatic intermittent titration technique (GITT). This testing protocol relies on Fick’s second law to extract the apparent diffusion coefficient. Because we are not measuring the change in concentration as a function of time or space, this method assumes all changes in concentration will be reflected by a voltage change based on the Nernst equation. By measuring voltage changes at various states of charge, it is possible to extract the apparent diffusion coefficient of ions in a solid.^48^ Figure 9 compares the apparent diffusion coefficient measured for both compounds at different lithiation states. Both materials show apparent diffusion coefficients between 10^–9^ and 10^–10^ cm^2^ s^–1^. These values suggest that both materials are considered good ionic conductors for Li–ions, and Li–ion diffusivity is not a limiting factor for these compounds. Several assumptions are required in extracting the apparent diffusion coefficient and small differences, for example, between values obtained for lithiation and for delithiation are not considered as significant here.

**Figure 9:**
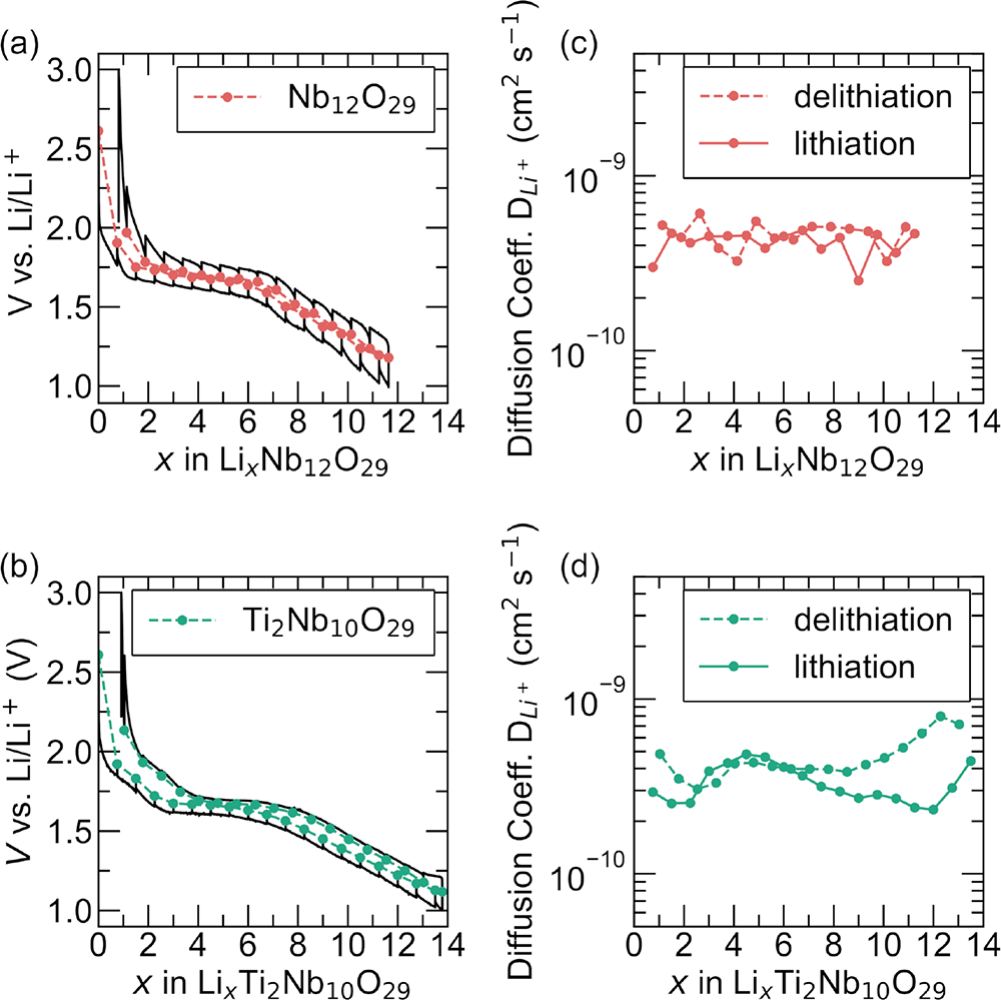
Raw data for the GITT measurements at C/10 charge and discharge for (a) Nb_12_O_29_ and (b) Ti_2_Nb_10_O_29_. The extracted apparent diffusion coefficient during (c, d) lithiation and delithiation of both compounds from GITT measurements.

Figure 10 plots the open-circuit voltage *U*_*OCV*_(*x*, *T*) and entropic potential ∂*U*_*OCV*_(*x*, *T*)/∂*T* of (a,c) Nb_12_O_29_ and (b,d) Ti_2_Nb_10_O_29_ coin cells as functions of lithium composition *x* during the first formation cycle. For the Nb_12_O_29_ coin cell, during the first lithiation half-cycle shown in Figure 10a, three distinct regions can be identified based on the evolution of *U*_*OCV*_ and ∂*U*_*OCV*_/∂*T*. In Region I, *U*_*OCV*_ decreases sharply with increasing *x*, while ∂*U*_*OCV*_/∂*T* features a rapid rise to a maximum followed by an equally swift drop. The corresponding increase in the electronic density of states and the electronic entropy lead to an increase in ∂*U*_*OCV*_/∂*T*.^47^ In Region II, *U*_*OCV*_ levels off slightly, but not to the extent of a plateau. Simultaneously, a significant tilde-shaped fluctuation appears in ∂*U*_*OCV*_/∂*T*. This is characteristic of lithium insertion in a homogeneous solid solution accompanied by intralayer ion ordering.^47^ Finally, Region III indicates an additional homogeneous solid solution regime with intralayer ion ordering, albeit less visible. This particular tilde-shaped fluctuation in ∂*U*_*OCV*_/∂*T* is smaller in magnitude and spans a narrower window of lithium composition. Theoretically, lithium ordering typically occurs between distinct regions within the crystallographic structure, and these data suggest that the difference in site energies for Li between the distinct regions is less pronounced than in Region II.^47^

**Figure 10:**
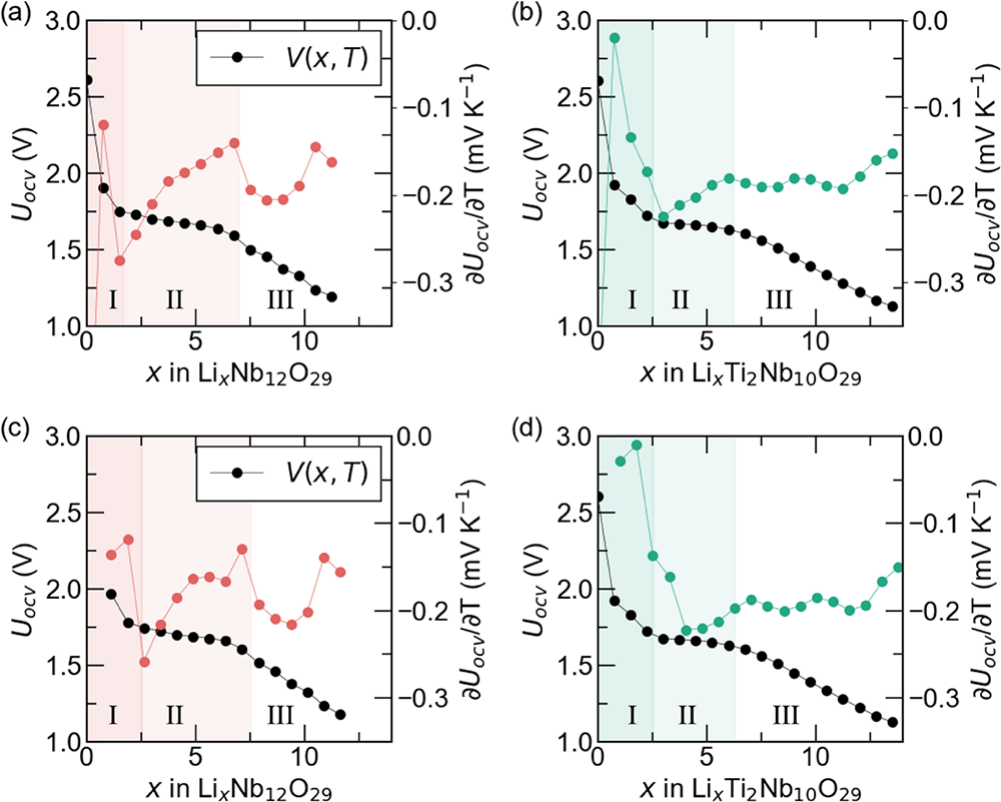
Open circuit voltage *U*_*OCV*_(*x*, *T*) and entropic potential ∂*U*_*OCV*_(*x*, *T*)/∂*T* of a Nb_12_O_29_ half cell as functions of lithium composition during (a) lithiation and (c) delithiation at C-rate of C/10. The same data for Ti_2_Nb_10_O_29_ during (b) lithiation and (d) delithiation.

An analogous pattern emerged for the Ti_2_Nb_10_O_29_ coin cell shown in Figure 10b. Region I is comparable to Nb_12_O_29_, suggesting that Ti_2_Nb_10_O_29_ also experiences a notable increase in electrical conductivity upon lithiation.^47^ By contrast, in Region II, Ti_2_Nb_10_O_29_ continues to show signs of homogeneous solid solution behavior with intralayer ion ordering. Furthermore, in Region III, *U*_*OCV*_ decreases monotonously, while ∂*U*_*OCV*_/∂*T* generally maintaining a consistent value, suggesting that the predominant charge storage mechanism is best described as only a homogeneous solid solution.^47^ Such distinctive intensity of lithium ordering around comparable lithium compositions in Regions II and III likely explains the divergent electrochemical performance for each material. As revealed by potentiometric entropy measurements, the charge storage mechanism in each region agreed with previous observations based on *operando* XRD measurements. Lastly, Figure 10c,d signify identical phenomena between the first lithiation and delithiation half-cycles for both Nb_12_O_29_ and Ti_2_Nb_10_O_29_.

The theoretical specific capacity of Nb_12_O_29_ is higher than that of Ti_2_Nb_10_O_29_ because of the two Ti transition metal substituting for Nb, with the lower mass. However, Ti can only undergo single electron redox, while each Nb can undergo two electron redox. Thus, larger capacities are expected for Nb_12_O_29_ compared to Ti_2_Nb_10_O_29_. However, this is not experimentally observed, which indicates that the Nb atoms in Ti_2_Nb_10_O_29_ must compensate by going to a lower oxidation state. To examine this hypothesis, we use X-ray photoelectron spectroscopy (XPS) and probe the oxidation states of the transition metals in both materials before and after Li insertion. Figure 11 shows the 3d Nb binding energy of the four samples and the oxidation states that are determined by duplicating the XPS spectra. The oxidation state assignments were made using both literature references as well as Nb_2_O_5_ reference collected on the same spectrometer. The duplication of peaks arises from spin–orbit splitting—the Nb 3d states split into Nb (lower binding energy) and Nb (higher binding energy). The results show that both pristine materials display their expected oxidation states. Nb_12_O_29_ shows a mixture of roughly 5:1 Nb^5+^ and Nb^4+^, while Ti_2_Nb_10_O_29_ contains only Nb^5+^. At 1 V, both Nb_12_O_29_ and Ti_2_Nb_10_O_29_ contain mostly a mixture of Nb^3+^ and Nb^4+^. The fact that Ti_2_Nb_10_O_29_ contains a higher proportion of Nb^3+^ (38%) compared to that in Nb_12_O_29_ (24%) can explain the similar capacity of the two compounds. In addition, we consider that the high percentage of Nb^3+^ in Ti_2_Nb_10_O_29_ may promote compound degradation, since Nb^3+^ is known to prefer trigonal-prismatic geometry in crystal structures,^57^ with the d^2^ configuration adopting a single, filled, low-lying d_*z* ^2^_ orbital. Such coordination is not normally possible in the structure types considered here. While this hypothesis requires further study, it is evident from long-term cycling data for Ti_2_Nb_10_O_29_ that capacity decreases as the number of cycles increases, which is different from Nb_12_O_29_, where less Nb is converted to Nb^3+^.

**Figure 11:**
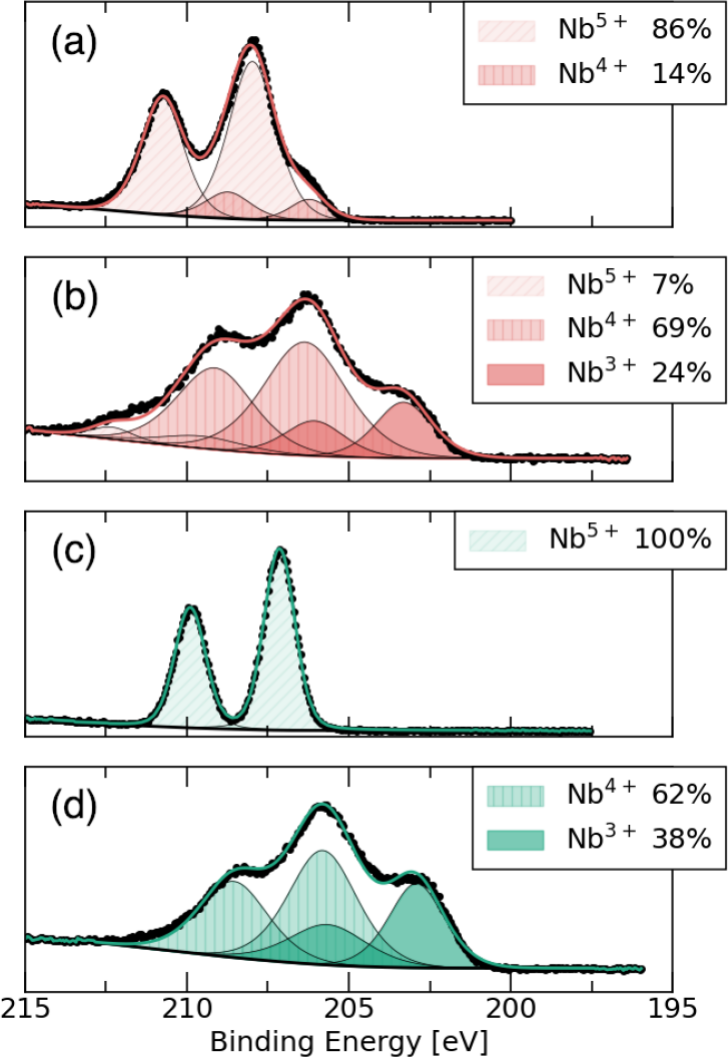
XPS measurements of pristine (a) Nb_12_O_29_ and (b) Li_14_Nb_12_O_29_ formed after charging to 1 V in the region of the Nb 3d binding energy. (c, d) Nb 3d XPS data of pristine Ti_2_Nb_10_O_29_ and Li_14_Ti_2_Nb_10_O_29_ samples charged up to 1 V. The lithiated samples were measured ex situ. Points represent the collected data, and the solid colored lines on each spectrum are the total fit arising from the peaks fitted to oxidation states. The area fraction of each oxidation state is noted in the legend.

## Conclusions

The materials studied here are promising anodes for fast charging Li–ion-based energy storage because of their high capacities and structural stability in addition to the excellent rate performance they display. By studying the role of disorder and initial metallicity in these materials, we can better understand and design electrochemical energy storage in these systems. Comparison between Nb_12_O_29_ and Ti_2_Nb_10_O_29_ suggests that the initial metallicity in Nb_12_O_29_ is not significantly advantageous over insulating Ti_2_Nb_10_O_29_ because of the rapid metallization of the latter with upon Li insertion, and this appears to be sufficient to ensure good electronic conductivity. Ti_2_Nb_10_O_29_ displays better rate performance than Nb_12_O_29_ because the transition metal disorder in Ti_2_Nb_10_O_29_ suppresses Li–ion ordering, leading to a solid solution intercalation mechanism. Detailed entropic potential measurements show that Li-ordering processes create an entropic energetic barrier. Mitigating such ordering through transition metal disorder allows for more facile Li kinetics. This study emphasizes the impact of transition metal disorder on Li–ion ordering and points to exciting directions for the design of faster-charging electrode materials with insights that are applicable to all material classes.
